# Andrographolide, a natural anti-inflammatory agent: An Update

**DOI:** 10.3389/fphar.2022.920435

**Published:** 2022-09-27

**Authors:** Xiaohong Li, Weichen Yuan, Jibiao Wu, Jianhua Zhen, Qihui Sun, Minmin Yu

**Affiliations:** ^1^ First Clinical School of Medicine, Shandong University of Traditional Chinese Medicine, Jinan, China; ^2^ School of Life Sciences, Beijing University of Chinese Medicine, Beijing, China; ^3^ College of Traditional Chinese Medicine, Shandong University of Traditional Chinese Medicine, Jinan, China; ^4^ Shandong University of Traditional Chinese Medicine, Jinan, China; ^5^ Affiliated Hospital of Shandong University of Traditional Chinese Medicine, Jinan, China

**Keywords:** andrographolide, inflammatory diseases, anti-inflammation, immunomodulation, review

## Abstract

Botanicals have attracted much attention in the field of anti-inflammatory due to their good pharmacological activity and efficacy. *Andrographis paniculata* is a natural plant ingredient that is widely used around the world. Andrographolide is the main active ingredient derived from *Andrographis paniculata*, which has a good effect on the treatment of inflammatory diseases. This article reviews the application, anti-inflammatory mechanism and molecular targets of andrographolide in different inflammatory diseases, including respiratory, digestive, immune, nervous, cardiovascular, skeletal, and tumor system diseases. And describe its toxicity and explain its safety. Studies have shown that andrographolide can be used to treat inflammatory lesions of various systemic diseases. In particular, it acts on many inflammation-related signalling pathways. The future direction of andrographolide research is also introduced, as is the recent research that indicates its potential clinical application as an anti-inflammatory agent.

## 1 Introduction

In recent years, the incidence of inflammatory diseases has remained high, and patient quality of life has worsened. Botanicals have attracted much attention in anti-inflammatory research due to their good pharmacological activity and efficacy. Andrographis paniculata, also called the “King of Bitterness” for its exceedingly bitter properties, belongs to the genus Andrographis and is a popular traditional medicinal plant. It is widely distributed in Asian countries, such as India, China, Malaysia, and Sri Lanka. In traditional Indian and Chinese medicine, it has been used for more than 1,000 years to treat inflammatory diseases.

**FIGURE 1:**
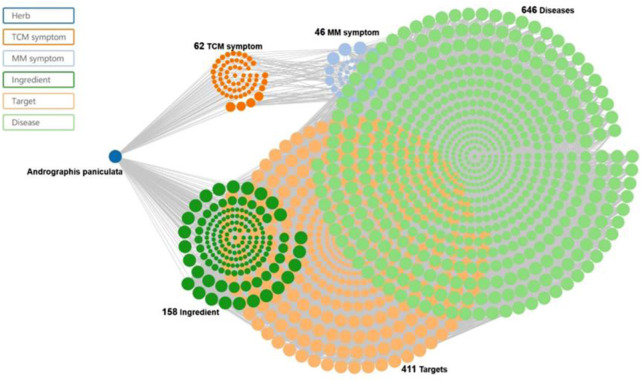
Traditional Chinese medicine (TCM) symptom-Chinese medicine-target network of the Chinese medicine. Andrographis paniculata and Chuan Xin Lian (SMHB00079), at 62 TCM symptoms, 46 Modern Medicine (MM) symptoms, 158 ingredients, 411 targets, and 646 diseases.

**FIGURE 2:**
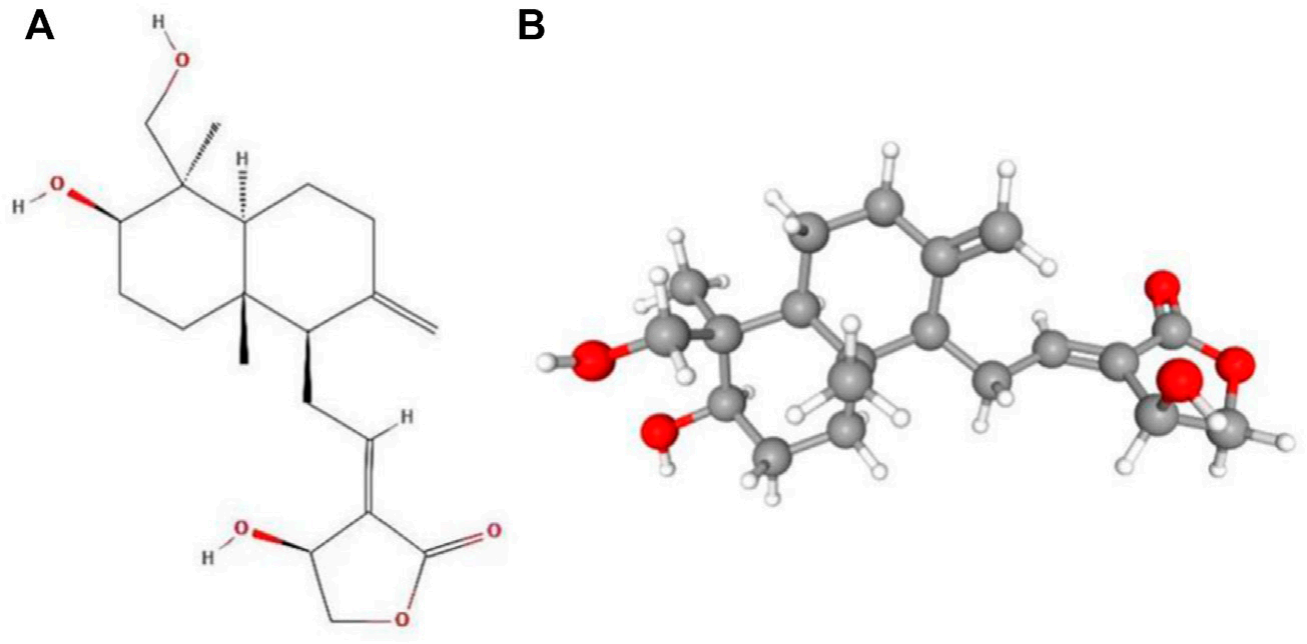
Molecular structure of andrographolide. **(A)** Two-dimensional structure of andrographolide. **(B)** Three-dimensional structure of andrographolide.

**FIGURE 3:**
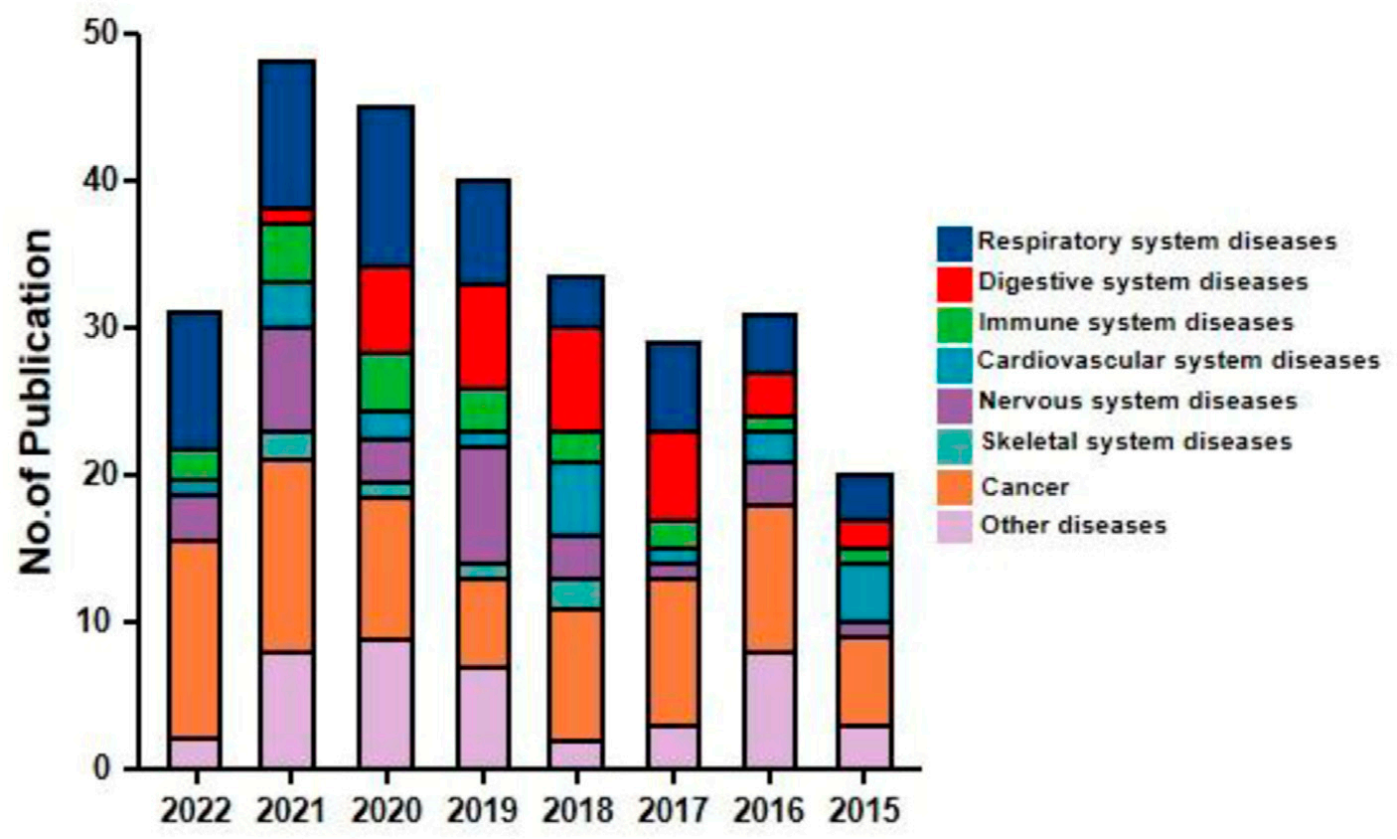
Number of publications on andrographolide (From 1 January 2015 to 1 August 2022)

**FIGURE 4:**
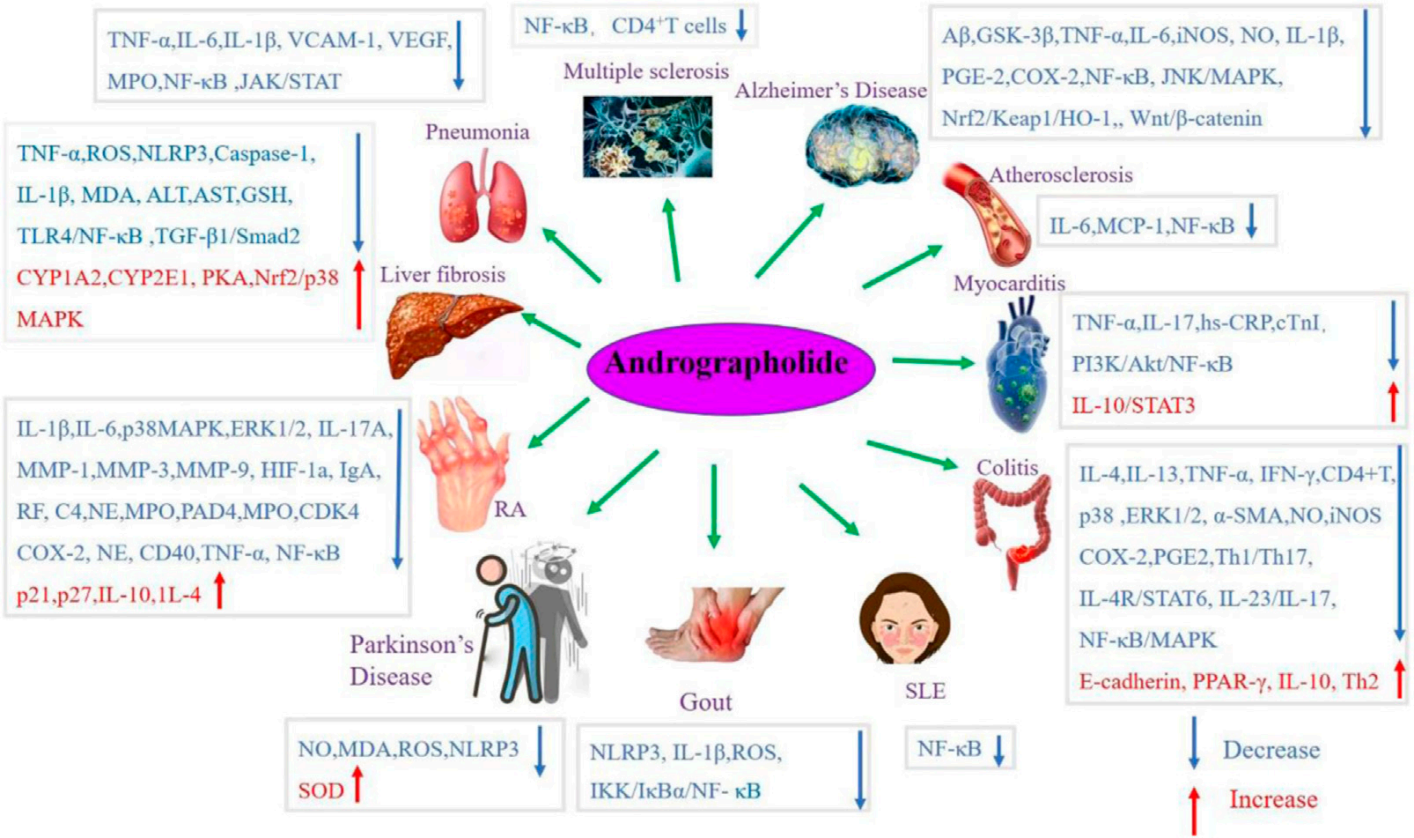
Andrographolide was used to treat inflammatory diseases.

Andrographolide (AD) was the main active substance that was isolated from the whole plant Andrographis paniculata in 1951 (Tan et al., 2017; Lu et al., 2019). Target network of Andrographis paniculata was predicted with SymMap (http://www.symmap.org/), the results were presented in (Figure 1 and Supplementary Tables S1, S2). It is a diterpene lactone compound, and its chemical formula is C_20_H_30_O_5_ (Figure 2). Previous research has confirmed that andrographolide has antipyretic and analgesic, anti-inflammatory, antibacterial, antiviral, immune regulatory, anti-tumor, neuroprotective, hepatoprotective, gallbladder protective, and anti-cardiovascular activities (Kishore et al., 2017). Recently, studies on andrographolide for treating inflammatory diseases have been increasing (Figure 3 is the literature on andrographolide published from 1 January 2015 to 1 August 2022), and it is widely used in China to treat inflammatory diseases and autoimmune diseases, including chronic obstructive pneumonia, liver fibrosis, rheumatoid arthritis (RA), systemic lupus erythematosus (SLE), etc. (Figure 4), showing that it has important anti-inflammatory value and application prospects. To the best of our knowledge, although there have been reviews of andrographolide in recent years, most are limited to single-system diseases and other pharmacological aspects, and there is no review of the anti-inflammatory effect of andrographolide on multisystem diseases. This article summarizes and reviews the research literature on andrographolide in the treatment of inflammatory diseases of different systems in the past 5 years and describes its mechanism of action and molecular targets. In particular, it summarizes the anti-inflammatory signaling pathways involved in its effects, discusses the evidence regarding the effectiveness and superiority of andrographolide in anti-inflammation, describes the shortcomings of andrographolide research at this stage and future research directions, and clarifies its clinical application prospects.

## 2 Application of andrographolide and its derivatives in inflammatory diseases

Andrographolide and its derivatives have good anti-inflammatory effects in different diseases, which are summarized as follows.

**TABLE 1:** Application and mechanism of andrographolide and its derivatives in inflammatory diseases.

Andrographolide and its derivatives	Inflammatory disease	Models	Mechanism	References
Andrographolide	Asthma	OVA-induced asthma mouse	Block the JAK1/STAT3 pathway to inhibit T17-related cytokines	Yu et al. (2021)
Andrographolide	Asthma	OVA-induced asthmatic mice	Inhibit NF-κB signalling, NLRP3 inflammasome and ROS	Peng et al. (2016a)
The 14-deoxy-11,12-didehydroandrographolide derivative, SRS27	Asthma	OVA-induced asthmatic mice	Inhibit NF-κB to reduce inflammatory response	Lim et al. (2016)
Andrographolide	COPD	Lipopolysaccharide-induced U937 cells, peripheral blood mononuclear cells (PBMCs) isolated from COPD model rats and patients	The inhibition of the PI3K/Akt/p70S6K signalling pathway and restored nuclear HDAC2 levels and activity	Liao et al. (2020)
Andrographolide	COPD	A mouse model of NTHi inflammation	The activation of Nrf2 and an increase in Keap1 levels	Tan et al. (2016)
Andrographolide	COPD	RAW264.7 cells induced by CS extract	Inhibit the SIRT1/ERK signalling pathway	Zhang et al. (2020)
Andrographolide	COPD	COPD mice induced by CS, human bronchial epithelial cells	IL-6/STAT3 signalling pathways	Xia et al. (2019)
Andrographolide	ALI	LPS-induced ALI mice	inhibit the MAPK and NF-κB pathways	Peng et al. (2016b)
				Nie et al. (2017)
Andrographolide	ALI	C57BL/6 mice	inhibit the transport of AIM2 to the nucleus to detect DNA damage	Gao et al. (2019)
Andrographolide	ALI	CS-induced ALI mice	Inhibit inflammatory cells and antioxidant	Guan et al. (2013)
3-Dehydroandrographolide	ALI	murine macrophage RAW 264.7 cells and BALB/c mice	activate the cholinergic anti-inflammatory pathway and inhibit the NF-κB/Akt pathway	Lu et al. (2018)
Andrographolide	Pulmonary fibrosis	silica-induced pulmonary fibrosis in male Swiss albino mice	prevent EMT by affecting fibroblasts	Karkale et al. (2018)
Andrographolide	Pneumonia	—	inhibit major protease activity	Shi et al. (2020)
Andrographolide	Pneumonia	THP-1 cells, C57BL/6 mice	Through NF-kB and JAK-STAT signalling pathways	Ding et al. (2017)
Andrographolide	ALI	male BALB/c mice (16–20 g)	Inhibit NF-kB pathway	Zhu et al. (2013)
AG-β-CD	Pneumonia	Male Sprague−Dawley (SD) rats (190–200 g)	immune regulation	Zhang et al. (2017a)
Andrographolide	Colitis	male Sprague Dawley rats with ulcerative colitis induced by oxazolinone	block the IL-4R-STAT6 pathway	Zhang et al. (2019a)
Andrographolide	Colitis	a mouse model of chronic colitis	decrease inflammation and epithelial damage as well as fibrosis	Gao et al. (2020)
Andrographolide	Colitis	experimental colitis mouse model	restrain the activation of the IL-23/IL-17 axis and the production of downstream proinflammatory cytokines	Zhu et al. (2018a)
Andrographolide	Colitis	DSS-induced acute colitis in mice	inhibit the NF-κB, MAPK and AMPK pathway	Kim et al. (2019)
Andrographolide	Colitis	PBMCs	inhibit the Th1/Th17 immune response and promote the Th2 response	Zhu et al. (2018b)
CX-10	Colitis	DSS-induced ulcerative colitis mice	inhibit the activation of NF-κB and MAPK pathways	Gao et al. (2018)
3b	Colitis	DSS-induced experimental colitis mice	inhibiting TLR4-NF-κB and enhancing β-catenin signaling pathway	Guo et al. (2019)
AL-1	Colitis	TNBS-induced colitis mice	Inhibit NF-κB pathway and promote PPAR-γ pathway	Yang et al. (2016)
AL-1	Colitis	DSS-induced colitis mice	inhibit NF-κB and MAPK signaling pathways	Jing et al. (2019)
Andrographolide	Toxic liver disease	mouse hepatocytes, Sprague–Dawley rats	increase the activity of liver microsomal enzymes (CYP1A2 and CYP2E1)	Jaruchotikamol et al.(2007)
				Akowuah et al. (2009)
Andrographolide	Toxic liver disease	Male C57BL/6J mice (25–30 g)	the suppression of inflammation and oxidative stress mediated by HO-1	Ye et al. (2011)
Andrographolide	Toxic liver disease	APAP-induced liver fibrosis mice	decrease Nrf2 activity and downstream antioxidant gene expression	Yan et al. (2018)
Andrographolide	Toxic liver disease	HepG2 cell	Adenosine A2A receptors activate p38 MAPK, upregulate Nrf2 expression	Mittal et al. (2016)
Andrographolide	Toxic liver disease	lipopolysaccharide/D-galactosamine-induced liver damage mice	suppress NF-κB and activate the Nrf2 signalling pathway	Pan et al. (2017)
Andrographolide	Liver Fibrosis	the liver of alcohol-exposed mice	reduce the expression of NF-κB and TNF-α, reduce pathological damage and oxidative stress	Song et al. (2020)
Andrographolide	Liver Fibrosis	the liver in mice with nonalcoholic steatohepatitis, high-fat HepG2 cells	reduce liver triglyceride content and macrophage infiltration, inhibit NF-κB activity and NLRP3 inflammasome	Cabrera et al. (2017)
Andrographolide	Liver Fibrosis	hepatic stellate cells	suppress TLR4/NF-κB and TGF-β1/Smad2 signalling pathways	Lin et al. (2018)
14-Deoxy-11,12-didehydroandrographolide	Liver Fibrosis	high-fat, high-cholesterol diet-induced fatty liver mice	Inhibit NF-κB signaling pathway	Liu et al. (2020)
Andrographolide	Osteoarthritis	elephant articular chondrocytes	repress the MAPK pathway and p38, ERK and JNK phosphorylation	Sirikaew et al. (2019)
Andrographolide	Osteoarthritis	mouse chondrocytes	regulate the inhibitory function of miR-27-3p on MMP-13	Chen et al. (2020)
Andrographolide	Osteoarthritis	FLSs cells isolated from the synovial tissue of rats and osteoarthritis patients	Inhibit NF-κB signalling pathway	Wang et al. (2021a)
Andrographolide	Osteoarthritis	an osteoarthritis mouse model and chondrocyte model	inhibit circ_Rapgef1/miR-383-3p/NLRP3	Yan et al. (2022)
Andrographolide	Rheumatoid Arthritis	Fibroblast-like synovial cells	upregulate the average levels of the cell cycle inhibitors p21 and p27 and reduce the content of cyclin-dependent kinase 4, increase mitochondrial cytochrome release and promote the activation of caspase-3	Yan et al. (2012)
Andrographolide	Rheumatoid Arthritis	Fibroblast-like synovial cells	inhibit the MAPK pathway	Li et al. (2017)
Andrographolide	Rheumatoid Arthritis	fibroblast-like synoviocytes, fibroid synovial cells	inhibit MMP-1, MMP-3, MMP-9, HIF-1α	Li et al. (2015)
Andrographolide	Rheumatoid Arthritis	60 RA patients	Reduce rheumatoid factor, IgA and complement C4	Burgos et al. (2009)
Andrographolide	Rheumatoid Arthritis	mice with complete Freund’s adjuvant -induced arthritis	reduce the expression of PAD4	Li et al. (2019)
Andrographolide	Rheumatoid Arthritis	CFA-induced arthritis models	Anti-inflammatory and antioxidant	Li et al. (2018a)
				Luo et al. (2020)
				Gupta et al. (2020)
Andrographolide	Gout	LPS-induced bone marrow macrophages and monosodium urate -induced mouse arthritis models	Suppress IKK, IκBα and NF-κB phosphorylation, inhibit IL-1β release, ROS, NLRP3 inflammasome and cysteine Asparaginase-1, induce HO-1 expression	Lo et al. (2021)
Andrographolide	Multiple sclerosis	mouse dendritic cells	inhibit NF-κB activation	Iruretagoyena et al. (2006)
Andrographolide	SLE	SLE mice	inhibit NF-κB activation	Kalergis et al. (2009)
Andrographolide	Myocarditis	myocardial tissue in autoimmune myocarditis rats	Suppress PI3K/Akt pathway	Zhang et al. (2019b)
Andrographolide	Myocarditis	viral myocarditis mice serum	Activate IL-10/STAT3 anti-inflammatory pathway, inhibit the PI3K/AKT/NF-κB pathway	Zhao et al. (2018)
Andrographolide micelles by PEG-PPS	atherosclerosis	LPS-activated macrophages	inhibit NF-κB pathway and ROS	Wu et al. (2018a)
Andrographolide	atherosclerosis	the murine macrophage cell line RAW264.7	inhibit NF-κB pathway	Wu et al. (2018b)
Andrographolide	coronary heart disease	rats with coronary heart disease	regulate PPAR and NF-κB signalling pathways	Shu et al. (2020)
Andrographolide	Obesity	obese mice	reverse the death receptor-dependent apoptosis pathway and mitochondria-dependent apoptosis pathway, enhance the IGF1R/PI3K/Akt signalling pathway	Hsieh et al. (2016)
Andrographolide	Alzheimer’s disease	AβPPswe/PS-1 transgenic Alzheimer’s mouse model	reduce β-amyloid levels	Serrano et al. (2014)
Andrographolide	Alzheimer’s disease	streptozotocin-induced Alzheimer’s disease rats	inhibit neuroinflammation	Patel et al. (2021)
Andrographolide	Alzheimer’s disease	mouse hippocampal HT22 cells	activate the Nrf2/Keap1-mediated HO-1 signalling pathway, inhibit the activation of Aβ42-overexpressing microglia BV-2, downregulate the NF-κB signalling pathway, reduce inducible nitric oxide synthesis	Seo et al. (2017)
Andrographolide	Alzheimer’s disease	primary microglia and BV-2 cells	inhibit the nuclear translocation of NF-κB	Yang et al. (2017a)
Two compounds of andrographolide	Alzheimer’s disease	Rat pheochromocytoma PC12 cell	Increase anti-inflammatory cytokines, decrease pro-inflammatory cytokines	Xu et al. (2019)
Andrographolide	Depression	chronic unpredictable mild stress mice	promote the expression of LC3 II and Beclin1, induce autophagy	Geng et al. (2019)
Andrographolide	Parkinson’s Disease	midbrain neuron-glia cocultures	Inhibit inflammatory mediators released by microglial activation and ROS production	Wang et al. (2004)
Andrographolide	Parkinson’s Disease	N9 mouse microglia (RRID CVCL_0452) cell line, C57BL/6 male mice	attenuate activation of the NLRP3 inflammasome	Ahmed et al. (2021)
Andrographolide and lipoic acid synthetic compounds	Parkinson’s Disease	MPTP-induced Parkinson’s mouse model	inhibit the loss of tyrosine hydroxylase (TH) positive neurons, increase the striatal expression of dopamine and its metabolite 3,4-dihydroxyphenylacetic acid, reduce the expression of nitric oxide and MDA	Zhang et al. (2014)
Andrographolide	Brain Injury	an intracerebral haemorrhage mouse model	inhibit the nuclear translocation of p65 and the assembly of the NLRP3/ASC/CASP-1 complex and inhibit the activation of NF-κB and NLPR3 inflammasomes	Li et al. (2018b)
Andrographolide	Brain Injury	a weightlessness-injured rat model	block the NF-κB and MAPK signalling pathways	Tao et al. (2018)
Andrographolide	Brain Injury	primary brain endothelial cells and rat brain tissue	regulate the p38 MAPK-Nrf2-HO-1 cascade	Yen et al. (2016)
Andrographolide	Brain Injury	a rat model of middle cerebral artery occlusion (up to 50% of the infarct size)	inhibit NF-κB and microglial activation, inhibit inflammatory factors	Chan et al. (2010)
Andrographolide	Brain Injury	mice with cerebral ischaemia/reperfusion	impede the activity of NF-κB and HIF-1α, reduce the production of NOX2 and iNOS	Chern et al. (2011)
Andrographolide derivatives	Brain Injury	Mouse macrophage RAW264.7 cells, Male BALB/c mice, Sprague-Dawley rats	inhibit TLR4/NF-κB signaling pathway and activate of Nrf2/ARE signaling pathway	Yang et al. (2019)
Andrographolide	Schizophrenia	a phencyclidine-induced mouse model of schizophrenia	activate the NRF-2 signalling pathway and inhibit the MAPK and NF-kB signalling pathways	Wang et al. (2021b)
Andrographolide	Chronic cerebral hypoperfusion	rats with chronic cerebral hypoperfusion	enhance the expression of BDNF and TrkB, and upregulate the BDNF-TrkB signalling pathway, lower the expression of TNF-α, IL-1β and Caspase-3	Wang et al. (2019)
Andrographolide	Working memory impairment	Mixed glial cells, adult BALB/c male mice	reduce the activity of NF-κB	Das et al. (2017)
Andrographolide	Intervertebral disc degeneration	nucleus pulposus cells	Block NF-κB pathway and reduce inflammatory factors	Liu et al. (2019)
Andrographolide	osteoporosis	mouse pre-osteoblastic (MC3T3-E1) cells, the estrogen-deficient (ovariectomized, OVX) rats	block RANKL-induced NF-κB and NFATC1 signalling pathways	Tantikanlayaporn et al. (2020)
Andrographolide	colon cancer	mice against affected by azoxymethane/dextran sulfate sodium-induced colon cancer	inhibit the PIK3CA-AKT1-MTOR-RPS6KB1 pathway	Guo et al. (2014)
Andrographolide	cancer	B16F0 melanoma syngenic and HT-29 xenograft models	promote the production of IL-2 and lymphocytes	Rajagopal et al. (2003)
Andrographolide	melanoma	B16F-10 melanoma cells	inhibit the expression of granulocyte-macrophage colony-stimulating factors and pro-inflammatory cytokines, inhibit the activation of NF-κB and AP-1	Pratheeshkumar et al. (2012)
Andrographis extract and andrographolide	tumor	metastatic tumor-bearing mice	reduce the level of pro-inflammatory cytokines, increase IL-2 to stimulate NK cells and T cell-mediated immune response	Sheeja and Kuttan (2010)
Andrographolide	cervical cancer	cervical cancer HeLa cells	inhibit the level of iNOS	Pasha et al. (2021)
Andrographolide	malaria	a murine model of malaria infection	the regulation of serine/threonine kinase and GSK3β and inhibition of the NF-κB pathway	Hassan et al. (2019)
Andrographolide	idiopathic dermatitis	mast cells and mice with atopic dermatitis	Inhibit Caspase-1/RIP2/NF-κB activity and inhibit the inflammatory response	Li et al. (2016)
Andrographolide	psoriasis	imiquimod-induced mouse psoriasis	inhibit the generation of proinflammatory cytokines and induce the autophagic proteolysis of MyD88	Shao et al. (2016)
Andrographolide	endothelial cell inflammation	endothelial cell	reduce NO and iNOS expression, inhibit Keap1 expression and increase Nrf2 expression	Fu et al. (2021)
Andrographolide	periodontal disease	periodontal ligament fibroblasts	inhibit the activation of NF-κB and STAT3	Ambili et al. (2017)
Andrographolide	abdominal aortic aneurysm	abdominal aortic aneurysm mouse	downregulate NF-κB-mediated generation of proinflammatory cytokines and expression of α4 integrin	Ren et al. (2016)
Andrographolide	nerve injury	Male 30 g BalbC mice	Inhibit NF-κB	Wang et al. (2018)

## 3 Respiratory diseases

### 3.1 Asthma

A variety of cells and cellular components produce chronic airway inflammation and are often accompanied by increased airway reactivity, resulting in symptoms such as chest tightness, wheezing, and shortness of breath, which lead to asthma. One study showed that andrographolide could reduce the expression of IL-6, IL-17A, and IL-17F in mouse serum and bronchoalveolar lavage fluid (BALF) and alleviate the infiltration of neutrophils in lung tissue and airway remodelling, primarily by blocking the JAK1/STAT3 pathway to inhibit T17-related cytokines in the treatment of ovalbumin (OVA)-induced asthma (Yu et al., 2021). The study indicated that andrographolide alleviated the symptoms of lung inflammation in OVA-induced asthmatic mice through mechanisms such as reducing the total number of leukocytes, macrophages, lymphocytes and neutrophils in BALF and reducing IL-6, IL-4, TNF-α, and IL-1β levels in BALF and serum, which are processes that are related to reactive oxygen species (ROS) scavenging, inhibition of NF-κB signalling, and NLRP3 inflammasome inhibition (Peng et al., 2016a). In an experimental mouse model of OVA, the 14-deoxy-11,12-didehydroandrographolide derivative, SRS27, decreased the counts of total cells, eosinophils, macrophages, neutrophils and lymphocytes in BALF of mice by a dose-dependent manner (0.1, 0.3, 1, and 3 mg/kg), decreased inflammatory cytokines IL-4, IL-5, IL -13, and CCL11 levels, increased IFN-γ, and decreased Th2-type cytokine levels and serum IgE levels; the mechanism is to inhibit NF-κB to reduce inflammatory response, and it is speculated that Wnt/β-catenin and 5-lipoxygenase Pathway may also be its target (Lim et al., 2016).

### 3.2 Chronic obstructive pulmonary disease (COPD)

The occurrence of COPD is closely related to chronic bronchitis and emphysema, and the clinical manifestation is the progressive development of airflow limitation, which is related to the enhancement of chronic airway inflammation. After treatment with andrographolide, dexamethasone restored the anti-inflammatory effect on lipopolysaccharide-induced U937 cells to produce IL-8 and restored glucocorticoid sensitivity in peripheral blood mononuclear cells (PBMCs) isolated from COPD model rats and patients. The levels of cigarette smoke (CS)-induced proinflammatory cytokines, such as IL-36γ, IL-17A, IL-1β, IL-27, and TNF-α, in BALF were reduced, and the mechanism might have been mediated by the inhibition of the PI3K/Akt/p70S6K signalling pathway and restored nuclear HDAC2 levels and activity, which reduced nuclear c-jun levels while promoting nuclear levels of the antioxidant transcription factor Nrf2 and reducing oxidative stress to increase HDAC2 levels (Liao et al., 2020). Andrographolide at a dose of 10 mg/kg lowered macrophage and neutrophil counts and decreased the expression of TNF-α, IL-1β, CXCL/KC, 8-hydroxytryptamine, MMP-8 and MMP-9 in mouse BALF in a mouse model of nontyped Haemophilus influenzae (NTHi) inflammation with increased CS exposure; the mechanism involves an increase in the expression of haem oxygenase-1 (HO-1), glutathione reductase (GR), glutathione peroxidase-2 (Gpx-2), glutamate cysteine and other antioxidant enzymes through the activation of Nrf2 and an increase in kelch-like ECH-related protein 1 (Keap1) levels to reduce inflammation and treat COPD (Tan et al., 2016). Andrographolide, which ameliorates mitochondrial function and mitochondrial membrane potential, can alleviate COPD by inhibiting the SIRT1/ERK signalling pathway, which attenuates the mitochondrial dysfunction, inflammation and oxidative stress in RAW264.7 cells induced by CS extract and can reduce the levels of proinflammatory factors (TNF-α and IL-1β), ROS, HO-1, MMP-9, and MMP-12 (Zhang et al., 2020). Andrographolide can reduce IL-6 expression and reverse CS-induced epithelial-mesenchymal transition (EMT) and pulmonary dysfunction of human bronchial epithelial cells. It decreases the number of mononuclear cells and neutrophils in the BALF of mice, reduces IL-6 and IL-8 expression levels, and restores lung dysfunction and small airway remodelling in mice, possibly through IL-6/STAT3 signalling pathways for the treatment of COPD (Xia et al., 2019).

### 3.3 Acute lung injury (ALI)

Pneumonia is an important cause of ALI. Inhibiting inflammatory factors and reducing the inflammatory response are important methods for treating it. In one approach, andrographolide lowered the concentrations of cytokines such as TNF-α, IL-6, and IL-1β in BALF and serum, and in mice, the mechanism involved the inhibition of the MAPK and NF-κB pathways, which alleviated LPS-induced ALI (Peng et al., 2016b; Nie et al., 2017). Andrographolide can also substantially block the activation of the AIM2 inflammasome induced by radiation in macrophages and pyroptotic response *in vivo* to alleviate radiation-induced lung injury, which can be employed as a novel conservatory agent against lung injury. Its mechanism involves the effective inhibition of the transport of AIM2 to the nucleus to detect DNA damage caused by cellular radiation or chemotherapy drugs (Gao et al., 2019). Andrographolide significantly mitigated lung inflammation in mice with CS-induced ALI and reduced the total number of inflammatory cells, neutrophils, and inflammatory mediators, such as IL-1β, IP-10, MCP-1 and KC, in BALF. In addition, it suppressed the expression of the oxidative biomarkers 8-isoprostane and 3-nitrotyrosine in the lung tissue of CS mice and enhanced the activities of antioxidant enzymes glutathione peroxidase (GSH-Px) and GR (Guan et al., 2013).3-Dehydroandrographolide also activated the cholinergic anti-inflammatory pathway and subsequently inhibited the NF-κB/Akt pathway, of which α7nAchR was a potential target for its anti-inflammatory effect (Lu et al., 2018).

### 3.4 Pulmonary fibrosis

The proliferation of fibroblasts and the accumulation of a great quantity of extracellular matrix that is accompanied by inflammatory responses and tissue damage are important features of pulmonary fibrosis. Andrographolide can exert antioxidant and anti-nitrosative stress activities; decrease the expression of interstitial markers, such as N-cadherin, α-smooth muscle actin and vimentin; upregulate E-cadherin levels; reduce the levels of inflammatory cells, such as total macrophages, neutrophils and lymphocytes in BALF of mice; and can reduce IL-1β, IL-6, TNF-α, TGF-β1, and hydroxyproline levels in lung tissue; these findings suggest that it prevents EMT from exerting its anti-fibrotic effects by affecting fibroblasts (Karkale et al., 2018).

### 3.5 Pneumonia

In human lung epithelial cells (Calu3), andrographis paniculata extract and andrographolide exhibited strong anti-SARS-CoV-2 activity in a dose-dependent manner, with high safety and no obvious cytotoxicity (Sa-Ngiamsuntorn et al., 2021). Andrographolide can dock to the binding site of SARS-CoV-2 Mpro as a potential inhibitor of the major protease of SARS-COV-2 (Mpro) (Enmozhi et al., 2021). Andrographolide can bind to TNF and NFkB1 proteins to block the NFkB1 signaling pathway of TNF-induced cytokine storm in COVID-19 patients (Rehan et al., 2021). Andrographolide inhibited major protease (MPRO) activity in 2019-nCoV and severe acute respiratory syndrome coronavirus (SARS-CoV), impairing SARS-CoV and 2019-nCoV replication (Shi et al., 2020). In THP-1 cells infected with PR8 (1000 TCID50/mL), andrographolide obstructed the influenza A virus-induced inflammatory response in mice through the NF-kB and JAK-STAT signalling pathways, alleviated pathological changes in lung tissue, reduced viral load, reduced infection-induced inflammatory cytokine expression and improved mouse survival (Ding et al., 2017). By inhibiting NF-κB pathway, andrographolide can attenuate LPS-induced lung inflammation, oedema and ultrastructural changes; inhibit MPO activity; reduce immune cell infiltration; and decrease TNF-α, IL-6, IL-1β, vascular adhesion vascular cellular adhesion molecule-1 (VCAM-1) and VEGF expression (Zhu et al., 2013). Andrographolide-β-cyclodextrin inclusion complex (AG-β-CD) treated *Staphylococcus aureus* pneumonia by reducing neutrophils, leukocytes, and total protein in BALF, reducing TNF-α, IL-6, NF-κB p65 expression and reduction of bacterial colonies (Zhang et al., 2017a).

The above description proves that andrographolide and its derivatives have good curative effect in respiratory system diseases, especially pulmonary inflammatory diseases such as asthma and chronic obstructive pulmonary disease. In pulmonary diseases, andrographolide has a large number of animal and cell experimental models for the treatment of inflammatory diseases, showing its important role in the treatment of pulmonary diseases. Its main mechanism is to directly inhibit the production of inflammatory cytokines and the activation of related pathways. However, there are few clinical trials, and the mechanisms involved are complex and ununified, and further exploration is needed.

## 4 Digestive system diseases

### 4.1 Colitis

Colitis is an inflammatory disease of the colon caused by various factors that can involve a variety of inflammatory cells. Andrographolide can block the IL-4R-STAT6 pathway in the colon tissue of mice with ulcerative colitis, effectively inhibit the oxazolinone-induced inflammatory response; reduce inflammatory cell infiltration; and decrease the levels of IL-4, IL-13, and TNF-α (Zhang et al., 2019a). In a mouse model of chronic colitis, andrographolide reduced CD4^+^ T-cell and macrophage infiltration in colonic tissue, reduced Th17 and Th1 differentiation, decreased p38 and ERK1/2 activation in colonic tissue, and reduced colonic tissue in mice with chronic colitis activation of MAPK and NF-κB; moreover, andrographolide increased E-cadherin levels and decreased α-SMA expression to alleviate colonic fibrosis (Gao et al., 2020). Andrographolide can restrain the activation of the IL-23/IL-17 axis and the production of downstream proinflammatory cytokines, thereby inhibiting the inflammatory response; reducing the levels of serum proinflammatory factors, such as TNF-α, IL-1β, IL-6, and IL-23; and inhibiting the Th17-cell immune response in patients with ulcerative colitis (Zhu et al., 2018a). Andrographolide can lower the expression of NO and proinflammatory cytokines, such as IL-6, TNF-α, and IL-1 to alleviate dextran sulphate sodium salt (DSS)-induced acute colitis in mice, and its mechanism is associated with the inhibition of the NF-κB and MAPK pathways in colon tissue and the activation of the AMPK pathway (Kim et al., 2019). Andrographolide is able to lower the expression levels of IFN-γ, IL-23, and IL-17A in the peripheral blood mononuclear cells (PBMCs) of patients with ulcerative colitis, increase the expression of IL-4, inhibit the Th1/Th17 immune response and promote the Th2 response (Zhu et al., 2018b). CX-10,andrographolide derivative, significantly reduced the expression of TNF-α and IL-6 and MPO activity in mouse colon tissue, decreased colon tissue damage, alleviated NF-κB p65 and p-IκBα, increased the expression of IκBα, and down-regulated the phosphorylation of p38 MAPK, ERK and JNK, which demonstrated that CX-10 can alleviate DSS-induced ulcerative colitis in mice by inhibiting the activation of NF-κB and MAPK pathways (Gao et al., 2018). Andrographolide derivative,3b, ameliorated DSS-induced experimental colitis in mice by inhibiting TLR4-NF-κB and enhancing β-catenin signaling pathway, which could reduce the level and transcription of pro-inflammatory cytokines in serum, while up-regulating β-catenin negatively regulated the immunosuppressive activity of 3b and enhanced TNF-α-mediated cell death (Guo et al., 2019). Andrographolide derivative,AL-1, which can significantly reduce proinflammatory cytokines TNF-α, IL-1β, and IL-6 level ameliorated TNBS-induced colitis in mice by down-regulating NF-κB pathway and up-regulating PPAR-γ pathway and suppressed inflammatory responses by reducing inflammatory cytokine levels and MPO activity (Yang et al., 2016). AL-1 ameliorated DSS-induced colitis in mice by inhibiting NF-κB and MAPK signaling pathways, reduced iNOS, COX-2, NO and PGE2 in cultured macrophages, significantly reduced production of IL-1β, IL-6, TNF-α, PGE2 and IFN-γ, and increased secretion of IL-10 (Jing et al., 2019).

### 4.2 Toxic liver disease

Liver diseases are mostly chronic inflammatory and autoimmune diseases, and inflammatory mediators or inflammatory factors are crucial in the pathogenesis of liver diseases. Their mechanisms involve oxidative stress and oxidase proliferation. Andrographolide targets various hepatotoxic substances by increasing the activity of liver microsomal enzymes (CYP1A2 and CYP2E1), maintains the stability of detoxification enzymes, and attenuates oxidative stress and cholestasis (Jaruchotikamol et al., 2007; Akowuah et al., 2009). Andrographolide improved histopathological changes, reduced TNF-α content, and alleviated carbon tetrachloride (CCl4)-induced hepatotoxicity, including reducing serum alanine aminotransferase, aspartate aminotransferase, and malondialdehyde (MDA) levels and crucially increasing glutathione levels, which may be relevant to the suppression of inflammation and oxidative stress mediated by HO-1 (Ye et al., 2011). Andrographolide has a protective effect on APAP-induced liver fibrosis, attenuates hepatic oxidative stress damage by decreasing Nrf2 activity and downstream antioxidant gene expression, and attenuates hepatic stellate cell activation and hepatic collagen deposition (Yan et al., 2018). Andrographolide can prevent H_2_O_2_-induced hepatic HepG2 cell death while reducing ROS and lipid peroxidation levels. The mechanism may be that andrographolide interacts with adenosine A2A receptors through activation of p38 MAPK. The expression of Nrf2 is then upregulated; at the same time, andrographolide can activate adenylate cyclase to promote the formation of cAMP and activate protein kinase A (PKA) to promote glycogen synthase kinase-3β (GSK-3β) phosphate inactivation of HO-1, which weakens the negative regulation of Nrf2 by GSK-3β and maintains the continuous activation of HO-1 (Mittal et al., 2016). Andrographolide has a conservatory effect on lipopolysaccharide/D-galactosamine-induced liver damage, which reduces the levels of ALT, AST, MPO, IL-1β, and TNF-α in liver tissue and decreases ROS and MDA contents by suppressing NF-κB and activating the Nrf2 signalling pathway (Pan et al., 2017).

### 4.3 Liver fibrosis

Any liver injury will develop into liver fibrosis during the healing process, and if the condition persists for a long time, it can develop into liver cirrhosis. Andrographolide can reduce the expression of NF-κB and TNF-α; reduce pathological damage and oxidative stress in the liver of alcohol-exposed mice; alleviate alcoholic liver disease in mice; and improve the levels of serum transaminases, liver function, lipid accumulation and liver reactive oxygen species (Song et al., 2020). Andrographolide can reduce liver triglyceride content and liver macrophage infiltration and reduce the expression of proinflammatory and profibrotic genes in the liver in mice with nonalcoholic steatohepatitis. It can also inhibit NF-κB activity and the activity of the NLRP3 inflammasome in high-fat HepG2 cells to reduce liver inflammation and fibrosis (Cabrera et al., 2017). A study has shown that in hepatic stellate cells, andrographolide can effectively reduce liver inflammation and fibrosis by suppressing the activity of the TLR4/NF-κB and TGF-β1/Smad2 signalling pathways (Lin et al., 2018). 14-Deoxy-11,12-didehydroandrographolide partially ameliorated steatohepatitis, liver fibrosis and liver injury in high-fat, high-cholesterol diet-induced fatty liver disease by enhancing hepatic Nrf2-mediated downstream antioxidant enzyme activity and inhibiting NLRP3 inflammasome activation, which can down-regulate the expression of NLRP3, Caspase-1 and IL-1β, and this anti-inflammatory property may be achieved by inhibiting the NF-κB signaling pathway (Liu et al., 2020).

In digestive system diseases, andrographolide and its derivatives can treat colitis, toxic liver disease and liver fibrosis. It has a good inhibitory effect on colon inflammation, and the mechanism mainly involves the direct inhibition of inflammatory factors and related pathways. Its treatment of liver disease mostly protects and restores liver function by restoring the activity of detoxification enzymes to exert an anti-inflammatory effect. The problems in its research are still inconsistent mechanisms, diverse cell and animal models and few clinical trials, and further clinical trials are needed, especially for colitis.

## 5 Immune system diseases

### 5.1 Osteoarthritis

Osteoarthritis is closely related to degenerative diseases and autoimmune reactions, and its pathogenesis is mostly related to inflammatory factors and many apoptotic cells. A study has shown that andrographolide reduces the expression of proinflammatory cytokines, including IL-1β, TNF-α and IL-6 (including OSM cytokines), as well as MMP-3 and MMP-13, in elephant articular chondrocytes. It has therapeutic function in osteoarthritis and may also be relevant to the repression of the MAPK pathway and the inhibition of p38, ERK and JNK phosphorylation (Sirikaew et al., 2019). Andrographolide can alleviate IL-1miR-1β-induced apoptosis in mouse chondrocytes, and its molecular mechanism is associated with regulating the inhibitory function of miR-27-3p on MMP-13, suggesting that it can treat osteoarthritis (Chen et al., 2020). Andrographolide can downregulate the expression of proinflammatory cytokines, such as IL-1β, IL-6 and TNF-α, and downregulate the expression of TNFR2 in FLSs isolated from the synovial tissue of rats and osteoarthritis patients, thereby reducing the downstream phosphorylation of p65 in the NF-κB signalling pathway, repressing the activity of NF-κB, and mitigating osteoarthritis synovial inflammation (Wang et al., 2021a). Andrographolide can attenuate the inflammatory response in an osteoarthritis mouse model and chondrocyte model, and the mechanism may involve the inhibition of circular RNA (circRNA) Rap guanine nucleotide exchange factor 1 (circ_Rapgef1)/microRNA-383-3p (miR-383-3p)/Nod-like receptor pyrin domain 3 (NLRP3) signal transduction to inhibit osteoarthritis (Yan et al., 2022).

### 5.2 Rheumatoid arthritis

Fibroblast-like synovial cells may be a crucial target for treating RA. Andrographolide inhibits the proliferation of rheumatoid arthritis fibroblast-like synoviocytes by upregulating the average levels of the cell cycle inhibitors p21 and p27 and reducing the content of cyclin-dependent kinase 4 and can arrest the cell cycle in G0/G1. Moreover, andrographolide can induce apoptosis in fibroblast-like synoviocytes by increasing mitochondrial cytochrome release and promoting the activation of caspase-3 (Yan et al., 2012). Andrographolide significantly reduces the phosphorylation of p38 MAPK and ERK1/2, as well as IL-1β and IL-6 content in TNF-α-stimulated fibroblast-like synovial cells, which may treat RA by inhibiting the MAPK pathway (Li et al., 2017). Under hypoxic conditions, andrographolide can inhibit the migration and invasion of fibroblast-like synoviocytes and significantly inhibit the upregulation of MMP-1, MMP-3 and MMP-9 expression; under anoxic circumstances, andrographolide downregulates the expression and DNA-binding activity of HIF-1α in fibroid synovial cells (Li et al., 2015). A prospective and randomized placebo-controlled clinical study of 60 patients with RA revealed that andrographolide preparations were effective in relieving RA symptoms, including reductions in rheumatoid factor, IgA and complement C4, with good safety (Burgos et al., 2009). Andrographolide significantly lowered the levels of proinflammatory cytokines (TNF-α, IFN-γ, IL-6, and IL-17A) in the plasma of mice with complete Freund’s adjuvant (CFA)-induced arthritis while improving the levels of the anti-inflammatory cytokine IL-10. PAD4 can alleviate rheumatoid arthritis in mice by promoting neutrophil apoptosis and inhibiting neutrophil autophagy, and PAD4 is a potential therapeutic target for treating rheumatoid arthritis. It was confirmed that andrographolide reduced the expression of PAD4 (Li et al., 2019). Andrographolide combined with methotrexate has a certain effect on the treatment of CFA-induced arthritis and can significantly reduce the expression levels of proinflammatory cytokines (TNF-α, IL-6, and IL-1β) in serum, alleviate methotrexate-induced hepatocyte injury and enhance anti-inflammatory effects (Li et al., 2018a). Andrographolide alleviates CFA-induced rheumatoid arthritis by attenuating oxidative stress and preventing multinucleated cell infiltration in diseased joints and synovial tissue; in medium and high doses of andrographolide, the levels of TNF-α, IL-6, CXC chemokine ligand 2, joint elastase, and MPO were considerably decreased, and the levels of antioxidant enzymes superoxide dismutase, catalase, and glutathione were improved (Luo et al., 2020). Andrographolide treated CFA-induced arthritis by inhibiting the expression of a series of arthritis-related molecules, including COX-2, NF-κB, p-p38, CD40, TNF-α, IL-1β, and IL-6 (Gupta et al., 2020).

### 5.3 Gout

Inhibiting inflammation is crucial in the treatment of gout. In LPS-induced bone marrow macrophages and monosodium urate (MSU)-induced mouse arthritis models, andrographolide suppressed the activity of the NLRP3 inflammasome, and its mechanism in the treatment of gout mainly involved the downregulation of LPS-induced IKK, IκBα and NF-κB phosphorylation to inhibit IL-1β release, reduce NLRP3 and pro-IL-1β protein expression, induce HO-1 expression, reduce reactive oxygen species generation, reduce LPS/MSU-induced NLRP3 inflammasome assembly and cysteine Asparaginase-1 formation and attenuate MSU phagocytosis (Lo et al., 2021).

### 5.4 Multiple sclerosis

Multiple sclerosis (MS) is an autoimmune disease in the central nervous system, and its main pathogenesis is the dysfunction of dendritic cells, resulting in an immune response to myelin damage (Sie and Korn, 2017). Andrographolide can downregulate humoral and adaptive cellular immune responses; *in vitro*, andrographolide interferes with T-cell proliferation and cytokine release in response to allogeneic stimulation, influences dendritic cell maturation and suppresses antigen presentation to T cells; in addition, andrographolide can significantly reduce the immune response in mice and alleviate MS symptoms in mice with autoimmune encephalomyelitis by suppressing T-cell activation and antibody responses against myelin sheaths (Iruretagoyena et al., 2005). Andrographolide inhibits NF-κB activation in mouse dendritic cells, impairs dendritic cell maturation and reduces the ability to activate antigen-specific T cells. In addition, NF-κB-blocked dendritic cells are specific for myelin antigens and inhibit encephalomyelitis progression (Iruretagoyena et al., 2006). Andrographolide shows a potential role in reducing the progression of brain atrophy and disability in multiple sclerosis (Ciampi et al., 2020).

### 5.5 Systemic lupus erythematosus

Systemic lupus erythematosus is a multisystem damage disease. Acute and chronic inflammation and tissue damage are important components of the development of the disease. In SLE mice with deficiencies in the inhibitory receptor FcγRIIb that lead to the production of antinuclear antibodies and glomerulonephritis, andrographolide inhibited SLE susceptibility in mice by inhibiting NF-κB activity, preventing the progression of antinuclear antibodies and renal injury (Kalergis et al., 2009).

In the autoimmune system, andrographolide shows good anti-arthritic disease effects. Its treatment of arthritis is well-documented and reasonable. Although its treatment of arthritis is still in the stage of animal testing, its mechanism of action is mostly inhibiting the infiltration of inflammatory cytokines and regulating autoimmune cells to fight inflammation. Since its mechanism is relatively clear and there are many animal experiments, the next step is to determine the dose and preparation of andrographolide for large-scale clinical trials. However, the number of treatment models for gout, multiple sclerosis and systemic lupus erythematosus is small, and large-scale animal and cell model validation is still needed in the next step. The mechanism of action of these three diseases also needs further research and summary to find out the corresponding mechanism.

## 6 Cardiovascular diseases

### 6.1 Myocarditis

Myocarditis is a localized or diffuse inflammatory disease of the myocardium. Andrographolide treatment can reduce plasma TNF-α, IL-17 and myosin antibody levels, increase IL-10 levels, and reduce the infiltration of CD3^+^ and CD14^+^ positive cells in myocardial tissue in autoimmune myocarditis rats; the anti-inflammatory activity is relevant to the suppression of the PI3K/Akt pathway (Zhang et al., 2019b). Andrographolide may improve viral myocarditis by inhibiting the increase in serum TNF-α, hs-CRP and cTnI levels caused by viral myocarditis, activating the IL-10/STAT3 anti-inflammatory pathway, and inhibiting the PI3K/AKT/NF-κB pathway (Zhao et al., 2018).

### 6.2 Atherosclerosis and coronary atherosclerotic heart disease

The development of chronic inflammation is an important cause of atherosclerosis, and the inhibition of inflammatory mediators and oxidative stress is an important therapeutic measure for coronary heart disease. The assembly of andrographolide micelles by polyethylene glycol-polythiopropylene block copolymer (PEG-PPS) can effectively downregulate the protein expression levels of IL-6 and MCP-1 in LPS-activated macrophages, and its anti-inflammatory function involves inhibition of the NF-κB pathway; at the same time, it can reduce reactive oxygen species levels to reduce oxidative stress to treat atherosclerosis (Wu et al., 2018a). Andrographolide can alleviate atherosclerosis by inducing anti-inflammatory effects and reducing the formation of ROS and foam cells, and its anti-inflammatory function is through the inhibition of the oxidized low-density lipoprotein-induced macrophage proinflammatory molecule monocyte NF-κB signalling pathway, which can downregulate the expression of inflammatory factors such as IL-6 and MCP-1 (Wu et al., 2018b). Andrographolide alleviates myocardial injury, endothelial dysfunction and inflammatory response in rats with coronary heart disease by regulating PPAR and NF-κB signalling pathways. Different concentrations of andrographolide can reduce the serum levels of ET, TXA2, TNF-α, MCP-1, hs-CRP, and IL-1β, which alters macrophage phenotypes (Shu et al., 2020).

### 6.3 Obesity

In the case of obesity, the body’s adipose tissue can lead to an improvement in proinflammatory factors and the formation of reactive oxygen species through anti-inflammatory factors, which results in a chronic low-grade inflammatory state. Andrographolide treatment can reverse the death receptor-dependent apoptosis pathway and mitochondria-dependent apoptosis pathway in obese mice, inhibit myocardial collagen deposition, cardiomyocyte apoptosis and cardiac hypertrophy, and inhibit the expression of the inflammatory marker COX-2 while enhancing the IGF1R/PI3K/Akt signalling pathway associated with cell survival, which is a compensatory survival mechanism that may mitigate the deleterious effects induced by a high-fat diet (Hsieh et al., 2016).

It can be seen from the above that andrographolide also has a certain therapeutic effect on cardiovascular diseases, such as myocarditis, obesity, atherosclerosis and other diseases. However, the number of models of andrographolide for each disease of the cardiovascular system is small, which cannot effectively confirm its anti-inflammatory effect in cardiovascular system diseases. However, its mechanism mostly involves inhibiting the PI3K/AKT/NF-κB pathway to exert an anti-inflammatory effect, and the mechanism of action is relatively unified.

## 7 Nervous system diseases

Andrographolide, which has the potential to treat mental illness, can reduce amyloid β-protein (Aβ) aggregation and inhibit neuroinflammation and synaptic dysfunction to treat Alzheimer’s disease, and andrographolide can also inhibit Parkinson’s disease, multiple sclerosis and cognitive impairment caused by surgery or diabetes (Lu et al., 2019). Andrographolide, as an NF-κB inhibitor, is a promising drug for treating stroke (Yang et al., 2017a).

### 7.1 Alzheimer’s disease

Neuroinflammation and microglial activation are critical in the development of Alzheimer’s disease. Pathological changes in an AβPPswe/PS-1 transgenic Alzheimer’s mouse model showed that andrographolide reduced β-amyloid levels to improve the ontogenesis of amyloid plaques in the cortical and hippocampal regions of young mice and reduced β-amyloid oligomer-induced tau protein phosphorylation, restoring and protecting synaptic plasticity and synaptic protein-related spatial memory function in Alzheimer’s disease mice (Serrano et al., 2014). Andrographolide can also modulate other signalling pathways involving Akt, NF-κB and MAPK to inhibit the progression of Alzheimer’s disease (Godoy et al., 2014). In addition, andrographolide has been shown to suppress GSK-3β activity and activate the downstream Wnt/β-catenin pathway associated with Alzheimer’s dysfunction (Tapia-Rojas et al., 2015). In streptozotocin-induced Alzheimer’s disease rats, the levels of neuroinflammatory markers (TNF-α, IL-1β, and IL-16) in the andrographolide-treated group were significantly reduced, and inhibition of neuroinflammation was a therapeutically important pathway for neurodegeneration (Patel et al., 2021). Andrographolide treatment of Alzheimer’s disease can activate the Nrf2/Keap1-mediated HO-1 signalling pathway in mouse hippocampal HT22 cells; inhibit the activation of Aβ42-overexpressing microglia BV-2; downregulate the NF-κB signalling pathway that reduces the production of IL-6, IL-1β, PGE2, and NO; and reduce inducible nitric oxide synthesis in the microglial cell line BV-2 and cyclooxygenase II levels (Seo et al., 2017). Andrographolide can significantly protect neural cells from microglia-mediated Aβ toxicity; reduce the release of proinflammatory products such as TNF-α, IL-1β, NO, and PGE2; and downregulate the protein levels of microglia-induced nitric oxide synthase and COX-2, which is related to inhibiting the nuclear translocation of NF-κB by affecting IκB phosphorylation and attenuating Aβ (1–42)-induced JNK-MAPK hyperactivation (Yang et al., 2017b). Two compounds of andrographolide can effectively inhibit the production of lipopolysaccharide-induced NO and the expression of induced nitric oxide synthase, as well as the pro-inflammatory cytokines TNF-α and IL-6, which have anti-inflammatory activity against microglia, and can protect nerve cells by inhibiting the production of pro-inflammatory mediators (Xu et al., 2019).

### 7.2 Depression

Inflammatory cell levels are increased in patients with depression, and the increase in inflammatory cell levels can aggravate depression. At 5 mg/kg, andrographolide promotes the expression of LC3 II and Beclin1 in the prefrontal cortex of chronic unpredictable mild stress (CUMS) mice and reduces p62 and p-mTOR levels, which indicates that it induces autophagy, which can improve mouse depression and inhibit inflammatory responses in the prefrontal cortex of mice and decreases the expression of proinflammatory mediators and cytokines (NO, COX-2, iNOS, IL-1β, IL-6, and TNF-α), inhibition of NF-κB signalling (p-P65, p-IκBα) and NLRP3 inflammasome assembly (NLRP3, Asc, and Caspase-1) (Geng et al., 2019).

### 7.3 Parkinson’s disease

Parkinson’s disease is a neurodegenerative disease in which dopaminergic neurons in the substantia nigra are progressively lost, and it is more common in elderly individuals. A more important pathogenesis is currently believed to be an increase in neuroinflammatory responses in the brain, during which hyperactivated microglia generate a large number of inflammatory mediators, which in turn lead to damage to dopaminergic neurons in the substantia nigra of the midbrain (Qiao et al., 2018). Andrographolide inhibits inflammatory mediators released by microglial activation and ROS production, thereby attenuating LPS-induced dopaminergic neurodegeneration in midbrain neuron-glia cocultures (Wang et al., 2004). Andrographolide attenuates activation of the NLRP3 inflammasome in microglia to treat Parkinson’s disease (Ahmed et al., 2021). Andrographolide and lipoic acid synthetic compounds can prevent 1-methyl-4-phenylpyridinium (MPP+)-induced neurotoxicity of SH-SY5Y cells and primary cerebellar granule neurons, inhibit the loss of tyrosine hydroxylase (TH) positive neurons in 1-methyl-4-phenyl-1, 2, 3, 6-tetrahydropyridine (MPTP)-induced Parkinson’s mouse model, increase the striatal expression of dopamine and its metabolite 3,4-dihydroxyphenylacetic acid, and improve MPTP-induced behavioral disorders; in addition, it can significantly reduce the expression of nitric oxide and MDA in substantia nigra of MPTP mice, increase the expression of superoxide dismutase, and then prevent nerve injury (Zhang et al., 2014).

### 7.4 Brain injury

An inflammatory response occurs after a brain injury. Andrographolide attenuates brain injury secondary to intracerebral haemorrhage by inhibiting the nuclear translocation of p65 and the assembly of the NLRP3/ASC/CASP-1 complex and inhibiting the activation of NF-κB and NLPR3 inflammasomes; it reduces microglial activation and neuroinflammation in an intracerebral haemorrhage mouse model, resulting in extremely decreased levels of TNF-α and IL-6 (Li et al., 2018b). Andrographolide, which can reduce brain oedema and apoptosis in brain tissue; improves neurobehavioral function and treats brain injury after craniocerebral trauma; inhibits the activation of microglia and neuroinflammation by blocking the NF-κB and MAPK signalling pathways in a weightlessness-injured rat model; and decreases the expression of the proinflammatory cytokines TNF-α, IL-6, and IL-1β (Tao et al., 2018). Andrographolide enhances HO-1 expression in primary brain endothelial cells and rat brain tissue by regulating the p38 MAPK-Nrf2-HO-1 cascade; in a study of a rat model of middle cerebral artery occlusion, andrographolide enhanced HO-1 expression by decreasing the production of free radicals in brain tissue and reducing brain oedema and infarct area to mitigate brain injury in rats with middle cerebral artery occlusion (Yen et al., 2016). Andrographolide can significantly lower the infarct size in a rat model of middle cerebral artery occlusion (up to 50% of the infarct size), inhibit the levels of the inflammatory cytokines TNF-α, PGE2, and IL-1β in ischaemic brain regions, and inhibit NF-κB and microglial activation to reduce neurological impairment (Chan et al., 2010). Andrographolide can impede the activity of NF-κB and HIF-1α, reduce the production of NOX2 and iNOS, and alleviate neurological impairment in mice with cerebral ischaemia/reperfusion (Chern et al., 2011). After treatment with andrographolide derivatives, it can effectively reduce the cerebral infarction area, improve neurological function, and reduce motor injury; its molecular mechanism may be related to the inhibition of TLR4/NF-κB signaling pathway and the activation of Nrf2/ARE signaling pathway (Yang et al., 2019).

### 7.5 Schizophrenia

Anti-inflammatory drugs can ameliorate the symptoms of schizophrenia. In a phencyclidine-induced mouse model of schizophrenia, andrographolide blocked the interaction between NRF-2 and Keap1; reduced NRF-2 degradation; promoted the nuclear translocation of NRF-2; and reduced the levels of p-p65, p-IκBα, p-p38, and p-ERK1/2, which reduced the levels of prefrontal cortex proinflammatory cytokines, such as IL-1, TNF, IL-6, COX-2 and iNOS levels by activating the NRF-2 signalling pathway and inhibiting the MAPK and NF-kB signalling pathways to reduce inflammation, reduce oxidative stress, and improve schizophrenia-like behaviour (Wang et al., 2021b).

### 7.6 Chronic cerebral hypoperfusion

Chronic cerebral hypoperfusion induces the activation of inflammasome components in distinct brain regions. Andrographolide can impede the activation of astrocytes, lower the expression of glial fibrillary acidic protein, enhance the expression of brain-derived neurotrophic factor (BDNF) and TrkB, and upregulate the BDNF-TrkB signalling pathway. It has a protective effect on hippocampal neuronal apoptosis in rats with chronic cerebral hypoperfusion and prevents related cognitive dysfunction; inhibits neuroinflammation; and lowers the expression of TNF-α, IL-1β, and Caspase-3 (Wang et al., 2019).

### 7.7 Working memory impairment

Anti-inflammatory treatments may reduce working memory impairment. Andrographolide can treat glial cell-induced neuroinflammation and neurodegeneration in the prefrontal cortex, thereby alleviating working memory impairment by altering the expression of synaptic and memory impairment markers; andrographolide can also block LPS-induced amyloid production, beta-site amyloid precursor protein cleaving enzyme (BACE) generation and other neuroinflammatory responses that lead to cognitive dysfunction, and the reason for the inhibition of these memory impairment markers may be that it reduces the activity of NF-κB (Das et al., 2017).

In nervous system diseases, andrographolide can have good anti-Alzheimer’s disease, anti-brain damage and anti-Parkinson’s disease effects, which are mostly accomplished by protecting nerve cells and inhibiting inflammatory cell infiltration. The mechanism is mostly achieved through NF-κB, MAPK, Nrf2 signaling pathway and inhibition of inflammasome NLRP3. Therefore, the next experimental model can involve more of these pathways or inflammatory factors. In the remaining other diseases, although it reflects a certain anti-inflammatory effect, due to the limitation of the small sample size, the anti-inflammatory evidence is not completely reliable.

## 8 Skeletal system disease

In some skeletal system diseases, andrographolide can exert a certain anti-inflammatory effect to treat bone degeneration and promote bone formation. Andrographolide reverses the degradation of human nucleus pulposus cells and improves Intervertebral disc degeneration (IDD) by blocking the NF-κB pathway and reducing the expression of inflammatory factors, including MMP-13, MMP-3, COX2, and PGE2 (Liu et al., 2019). Andrographolide promotes the differentiation of mouse osteoblast precursor cells (MC3T3-E1) by increasing the activity of the osteogenic gene-specific marker alkaline phosphatase, promotes bone formation, and increases the production of bone structure genes; the promotion of osteoblast differentiation is related to the OPG/RANKL signalling pathway, and it has a strong anti-inflammatory function in osteoclasts by blocking RANKL-induced NF-κB and NFATC1 signalling pathways (Tantikanlayaporn et al., 2020).

We found few samples of andrographolide used in the skeletal system, which limits the study of its mechanism to a certain extent. Large-scale animal and cell experiments are needed to confirm its application value in the skeletal system.

## 9 Tumor

Chronic inflammation is closely related to the occurrence and metastasis of cancer. For example, under the stimulation of pro-inflammatory cytokines, transcription factors NF-κB and STAT3 can promote the expression of oncogenes to promote tumorigenesis and metastasis. Therefore, drugs targeting chronic inflammation and natural Products such as andrographolide have good anticancer prospects (Zhang et al., 2017b). Andrographolide and its derivatives have been proven to have a certain therapeutic effect on gastrointestinal tumors such as gastric cancer and intestinal cancer (Wang et al., 2021c). Andrographolide can also enhance the antitumor properties of antitumor drugs, for example, it can enhance the apoptosis of colorectal cancer cells after 5-Fu administration (Su et al., 2017). Inhibiting inflammation may reduce colitis-related cancers. A study have demonstrated that andrographolide triggers mitotic phagocytosis to selectively clear damaged mitochondria in cells by inhibiting the PIK3CA-AKT1-MTOR-RPS6KB1 pathway, resulting in a reverse collapse of mitochondrial membrane potential, which inactivates the NLRP3 inflammasome to protect mice against affected by azoxymethane/dextran sulfate sodium-induced colon cancer (Guo et al., 2014). A Study have shown that andrographolide can promote the production of IL-2 and lymphocytes, and IL-2 activates the cytotoxic activity of NK cells, CD8 + T cells and lymphokine-induced killer cells, and produces TNF-α, which increases the cytotoxicity of lymphocytes to cancer cells (Rajagopal et al., 2003). In B16F-10 melanoma cells, andrographolide inhibits the expression of granulocyte-macrophage colony-stimulating factors and pro-inflammatory cytokines such as TNF-α, IL-1β and IL-6 genes, inhibits the activation of NF-κB and AP-1 to promote apoptosis of tumor cells (Pratheeshkumar et al., 2012). Andrographis extract and andrographolide can effectively reduce the level of pro-inflammatory cytokines (such as IL-1β, IL-6, GM-CSF and TNF-α) in metastatic tumor-bearing mice to inhibit tumor metastasis, while increasing IL-2 to stimulate NK cells and T cell-mediated immune response to eliminate cancer cells (Sheeja and Kuttan, 2010). Inducible nitric oxide synthase (iNOS), as an inflammatory factor, is involved in mediating inflammation, and persistent inflammation can promote cell transformation, proliferation, invasion and angiogenesis in malignant tumors. A study has shown that andrographolide can inhibit the level of iNOS in cervical cancer HeLa cells, and andrographolide has anti-proliferative and pro-apoptotic properties on cervical cancer cells, but its specific pathways and mechanisms still need to be further explored (Pasha et al., 2021).

It can be seen from the above that inhibiting the expression of inflammatory factors to reduce the level of inflammation can reduce tumor metastasis and invasion to a certain extent, so anti-inflammatory has a certain therapeutic effect on cancer. As a natural product, andrographolide has a good anti-inflammatory effect and can also treat malignant tumors. Its mechanism of action mostly focuses on directly inhibiting inflammatory factors, and its specific pathway and mechanism of action need to be further explored.

## 10 Other inflammatory diseases

Andrographolide also has therapeutic effects in other inflammatory diseases. In a murine model of malaria infection, andrographolide significantly decreased the levels of proinflammatory cytokine IFN-γ and improved the levels of anti-inflammatory cytokines IL-10 and IL-4, and the mechanism may have partly involved the regulation of serine/threonine kinase and GSK3β and inhibition of the NF-κB pathway (Hassan et al., 2019). In mast cells and mice with atopic dermatitis, andrographolide can inhibit the expression of thymic interstitial lymphopoietin by inhibiting Caspase-1/RIP2/NF-κB activity and inhibit the inflammatory response to treat idiopathic dermatitis (Li et al., 2016). In imiquimod-induced mouse psoriasis, andrographolide inhibited the generation of proinflammatory cytokines such as IL-23 and IL-1b and induced the autophagic proteolysis of MyD88, which controlled MyD88-dependent cytokine activation and alleviated psoriasis (Shao et al., 2016). Andrographolide can reduce LPS-induced NO and iNOS expression, inhibit Keap1 expression and increase Nrf2 expression, thereby inhibiting proinflammatory factor expression to reduce endothelial cell inflammation (Fu et al., 2021). Andrographolide can treat periodontal disease by inhibiting the activation of NF-κB and STAT3 in periodontal ligament fibroblasts and inhibiting inflammation and bone resorption-related genes (Ambili et al., 2017). Andrographolide may inhibit the infiltration of inflammatory cells by downregulating NF-κB-mediated generation of proinflammatory cytokines (CCL2, CXCL10, TNF-α and IFN-γ) and expression of α4 integrin, thereby inhibiting abdominal aortic aneurysm progression (Ren et al., 2016). Andrographolide may reduce mechanical hypersensitivity in nerve injury models by antagonizing NF-κB to reduce inflammatory cytokines produced by the spinal cord, such as the proinflammatory cytokine IL-1 in the dorsal horn, and exert antialodynia effects (Wang et al., 2018).

## 11 Safety testing of andrographolide and its derivatives

Although andrographolide and its derivatives have good anti-inflammatory effects, they also have certain biological toxicity and adverse reactions. The results of a randomized controlled trial of 44 patients showed that andrographolide in the treatment of multiple sclerosis showed a trend to reduce the rate of brain atrophy and disability progression, but andrographolide had adverse events of rash and dysgeusia (Ciampi et al., 2020). Some people have pointed out that their nephrotoxicity and reproductive toxicity are more common (Zeng et al., 2022). In terms of hepatotoxicity, from the application of andrographolide in liver diseases, we can find that andrographolide has a good repairing effect on hepatotoxicity, but there is no evidence that it can induce liver damage. A study pointed out that the lethal dose of andrographolide-2-hydroxypropyl-β-cyclodextrin is greater than 2000 mg/kg, and it has no adverse effects on animal growth, circulating blood cells, and liver and kidney functions, and has good safety (Chandrama Singh et al., 2022). In terms of nephrotoxicity, andrographolide easily damages renal tubular epithelial cells and causes kidney damage. Andrographolide (0–250 μmol/L) inhibits human renal tubular epithelial (HK-2) cell proliferation and induces apoptosis in a dose- and time-dependent manner, accompanied by decreased superoxide dismutase activity and increased malondialdehyde content, and the mechanism of which may be related to endoplasmic reticulum stress and inflammatory response (Gu et al., 2016). Andrographolide sodium bisulfate (ASB) can induce autophagy in HK-2 cells with the prolongation of administration time (8–24 h) and the increase of drug concentration (7–57 mmol/L), and the cell viability and cell membrane integrity gradually decrease. The specific mechanism is that ASB can induce HK-2 cells to generate ROS, activate the JNK-mediated signaling pathway, and induce apoptosis through the caspase-dependent mitochondrial pathway, while ASB induces autophagy by enhancing the expression of Beclin-1 (Lu et al., 2014). Mitochondria are the main targets of ASB-induced nephrotoxicity (Xing et al., 2015). A study that chemically altered the structure of andrographolide to construct new andrographolide derivatives showed that most andrographolide derivatives were less nephrotoxic to HK-2 cells than andrographolide (Gu et al., 2021). In terms of reproductive toxicity, studies have shown that Andrographis paniculata has no dose-dependent effect on the reproductive toxicity of male Wistar rats, and the sperm quality and reproductive function of rats are not affected when the maximum dose is 1,000 mg/kg/day; at the same time, a phase I clinical study also confirmed that taking different doses of Andrographis paniculata had no significant effect on human fertility parameters (Allan et al., 2009). However, one study found that male Wistar rats with andrographis could reduce sperm count and motility, reduce serum testosterone levels, reduce xanthine oxidase and myelopectidase activity, increase testicular glutathione levels and superoxide dismutase activity after administration, indicating that it induces reproductive toxicity by suppressing testosterone rather than inducing oxidative stress, and speculating that it can be used as a safe male contraceptive (Ogundola et al., 2021).

It can be seen that the safety of andrographolide and its derivatives is high, and it is speculated that there is basically no obvious hepatotoxicity, and there is a certain degree of nephrotoxicity and reproductive toxicity, but renal toxicity is mostly related to dose and compound structure, and reproductive toxicity needs further research to confirm. At the same time, in order to reduce and avoid the side effects of related toxicity, the first is to carry out large-scale clinical experimental research, and strictly regulate the experimental dosage form, administration method, administration time, administration dose and sample size to ensure the reliability of clinical trials. The second is to test the pharmacokinetics of andrographolide and its derivatives to determine the appropriate dosage. The third is to change the chemical structure of andrographolide to synthesize new compounds with less toxicity. At present, most of its clinical trials have involved andrographolide preparations, such as Xiyanping injection, and there have been few clinical trials of andrographolide alone. A multicentre randomized controlled trial with a small sample found that Xiyanping injection had a significant effect on the treatment of pulmonary inflammation caused by mild to moderate COVID-19 pneumonia, with no obvious side effects and good safety (Zhang et al., 2021). Therefore, to a certain extent, a large-scale clinical safety study of andrographolide preparations can also confirm the safety of andrographolide, which may become the next research direction.

## 12 Conclusion

Because of the increasing incidence of inflammation worldwide, there is an urgent need to identify a highly effective anti-inflammatory drug. In this review, by reviewing the application and mechanism of andrographolide in the treatment of inflammatory diseases, we found that andrographolide has good anti-inflammatory and immunomodulatory effects and is a promising drug for inflammatory diseases. First, it has the effect of treating respiratory, digestive immune, cardiovascular, and nervous system diseases. It treats inflammatory diseases of multiple systems. The anti-inflammatory function has been explained in the context of a variety of systemic diseases, among which the respiratory system, digestive system, nervous system and immune system diseases have been widely studied. The anti-inflammatory function of andrographolide is mainly manifested in the treatment of asthma, chronic obstructive pulmonary disease and lung injury in respiratory system diseases and the treatment of colitis and liver diseases in the digestive system. Brain injury, Alzheimer’s disease and Parkinson’s disease are predominant neurological diseases, and rheumatoid arthritis is a predominant immune system disease. Andrographolide shows strong and well-documented anti-inflammatory effects in the above diseases, while the data regarding its anti-inflammatory effects in other diseases are limited, and further large-scale experimental verification and mining are needed. Second, andrographolide alleviates inflammatory diseases mainly by reducing the levels of inflammatory mediators such as TNF-α and IL-6 in cells and animal models, which exerts immunomodulatory and antioxidative stress effects. Third, andrographolide can exert anti-inflammatory effects through a variety of targets and signalling pathways, including blocking the NF-κB, MAPK, PI3K/Akt, NLRP3 and other pathways. Among them, the inhibitory effect on the NF-κB pathway has been the most studied, which strongly confirms the important role of the NF-κB pathway in the anti-inflammatory pathway of andrographolide. The next step is to use andrographolide in a variety of diseases to conduct specific experiments targeting the NF-κB pathway, determine the applied dose, and conduct large-scale data observation will become the focus of research. And by observing its specific pathways in different systems, it is possible to better carry out precise experiments and treatments for diseases of different systems. As mentioned above, it mostly acts through the PI3K/AKT/NF-κB pathway in the cardiovascular system. There are few research data on other mechanisms of action, and it is very challenging to mine new signalling pathways and screen the most effective signalling pathways. Fourth, the anti-inflammatory effects of other anti-inflammatory drugs and andrographolide are relatively small, and the combined application is also insufficient, so it is impossible to clearly determine the level of effectiveness of andrographolide. Therefore, animal or clinical trials of other anti-inflammatory drugs in combination with andrographolide should be performed to determine the level of efficacy of andrographolide. Finally, according to the safety test of andrographolide, determine the efficacy and safety level, understand its adverse reactions and side effects, reduce its toxicity, and promote it in clinical practice.

In conclusion, andrographolide has an anti-inflammatory effect, which has been confirmed by experiments in multiple systemic diseases. Further research is needed to explore the pharmacological mechanisms of andrographolide, its toxicity and adverse reactions, its pharmacokinetics and the optimal therapeutic dose. Clinical studies are needed to further apply the drug more precisely.

## Glossary

AD: Andrographolide
RA: Rheumatoid arthritis
SLE: Systemic lupus erythematosus
IL-17A: Interleukin-17A
IL-6: Interleukin-6
IL-17F: Interleukin-17F
BALF: Bronchoalveolar lavage fluid
JAK/STAT: The Janus kinase/signal transducer and activator of tran-scriptions
OVA: Ovalbumin
IL-4: Interleukin-4
IL-1β: Interleukin-1β
TNF-α: Tumor necrosis factor
ROS: Reactive oxygen species
NF-κB: Nuclear factor kappa B
NLRP3: NOD-like receptor thermal protein domain associated protein 3: NLR family, pyrin domain containing 3
COPD: Chronic obstructive pulmonary disease
IL-8: Interleukin-8
CS: Cigarette smoke
IL-27: Interleukin-27
IL-36γ: Interleukin-36γ
PI3K: Phosphatidylinositol 3-kinase
HDAC2: Histone deacetylase 2
Nrf2: Nuclear factor erythroid related factor 2
CXCL: CXC chemokine ligand
KC: Keratinocyte-derived chemokines
MMP: Matrix metalloproteinase
NTHi: Non-typed haemophilus influenzae
HO-1, Heme oxygenase-1
GR: Glutathione reductase
Gpx-2: Glutathione peroxidase-2
Keap1: Kelch-like ECH-related protein 1
SIRT1: Sirtuin1
ERK: Extracellular regulated protein kinases
EMT: Epithelial-mesenchymal transition
ALI: Acute lung injury
MAPK: Mitogen activated protein kinase
LPS: Lipopolysaccharide
AIM2: Absent in melanoma 2
IP-10: IFN-γ inducible protein 10
MCP-1: Monocyte chemoattractant protein-1
GSH-Px: Glutathione peroxidase
TGF-β1: Transforming growth factor-β1
MPRO: Major protease
SARS-CoV: Severe acute respiratory syndrome coronavirus
PR8: A/Puer to Rico/8/34
TCID50: Half the tissue culture infectious dose
MPO: Myeloperoxidase
VEGF: Vascular endothelial growth factor
VCAM-1: Vascular Cellular Adhesion Molecule-1
IL-4R: Interleukin-4R
Th17: T helper 17
Th1: T helper 1
α-SMA: Alpha-smooth muscle actin
IL-23: Interleukin-23
IL-17: Interleukin-17
NO: Nitric oxide
IL-1: Interleukin-1
DSS: Dextran sulfate sodium salt
AMPK: Adenosine 5‘-monophosphate (AMP)-activated protein kinase
IFN-γ: Interferon-γ
PBMC: Peripheral blood mononuclear cells
Th2: T helper 2
CCI4: carbon tetrachloride
MDA: Malondialdehyde
APAP: acetaminophen
H2O2: Hydrogen peroxide
cAMP: cathelicidin antimicrobial peptide
GSK- 3β: Glycogen synthase kinase-3β
AST: Aspartate aminotransferase
ALT: Alanine aminotransferase
PKA: Protein kinase A
TLR4: Toll Like Receptor 4
OSM: Oncostatin M
TNFR2: Tumor necrosis factor receptor-2
HIF-1α: HIF-1 alpha
IL-10: Interleukin-10
IgA: Immune globulin A
PAD4: Peptidylarginine deiminase 4
CFA: complete Freund’s adjuvant
COX-2: Cyclooxygenase 2
MSU: monosodium urate
IKK: IκBkinase
IκBα: NF-kappa-B inhibitor alpha
MS: Multiple sclerosis
FcγRIIb: Inhibitory IgG receptor
hs-CRP: high-sensitivity C-reactive protein
cTnI: cardiac troponin I
PEG-PPS: Polyethylene glycol-polythiopropylene block copolymer
PPARs: Peroxisome proliferators-activated receptors
TXA2: Thromboxane 2
ET: endothelin
IGF1R: Insulin Like Growth Factor 1 Receptor
Aβ: amyloid β-protein
PGE2: prostaglandin E2
COX-II: Cyclooxygenase II
CUMS: Chronic unpredictable mild stress
m-TOR: mammalian target of rapamycin
iNOS: Nitric oxide synthetase
CASP-1: Caspase 1
NOX2: NADPH oxidase 2
BDNF: brain-derived neurotrophic factor
TrkB: Tropomyosin-related kinase B
BACE: beta-site amyloid precursor protein cleaving enzyme
OPG: osteoclastogenesis inhibitory factor
RANKL: Receptor Activator of Nuclear Factor-κB Ligand
NFATC1: Nuclear Factor Of Activated T Cells 1
MyD88: myeloid differentiation factor 88
CCL2: Monocyte chemoattractant protein-1
MTOR: mechanistic target of rapamycin
NLRP3: NOD-like receptor thermal protein domain associated protein 3: NLR family, pyrin domain containing 3
AKT1: v-akt murine thymoma viral oncogene homolog 1.
