# Supplementary material for: Andrographolide, a natural anti-inflammatory agent: An Update
Source: Front Pharmacol. 2022 Sep 27;13:920435. doi: 10.3389/fphar.2022.920435 (PMC9551308; doi:10.3389/fphar.2022.920435)
Supplement: Supplementary file 1 [file DataSheet1.PDF]

Supplemental Tble 1 MM symptom name

MM symptom name
Pharyngolaryngitis
Blanching Of Skin
Larynx
Laryngopharyngeal Dysaesthesia
Tongue Ulcer
Localized; Swelling
Physical Suffering
Cough Productive
Laryngeal Pain
Catarrh
Cyst
Laryngeal Edema
Trachoma
Smarting Of Skin
Hyperalgia
Diarrhea
Skin Pain
Bite Wound Insect
Pain
Cough
Burning Pain
Splitting Pain
Venomous Bite
Diarrhea Acute
Migratory Pain
Carbuncle
Skin Ulcer(S)
Swelling
Diarrhea Watery
Coryza
Ache
Urinary Tract Pain
Throat Tightness
Dysentery
Snake Venom Causing Toxic Effect
Pain Radiating
Crushing Pain
Common Cold
Pharyngolaryngeal Pain
Aphthous Ulcer
Mental Status Altered

Chills And Fever
Edema Pharynx
Oral Ulcer
Sutton'S Disease
Fever

Supplemental Table 2 TCM symptom name

TCM symptom name
Chong Yao Zhong Tong
Chuang Yang
Di Re
Du She Yao Shang
Dun Ke
Fa Re
Fa Re Zhong
Fa Shao
Gan Mao
Han Xing Fu Xie
Hou He Hong Zhong
Hou He Zhong Da
Hou Tong
Hou Zhong
Ji Hou Bi
Ju Bu Zhong Tong
Kou Bi Sheng Chuang
Kou Chuang Zhong Tong
Kou Mi Lan
Kou Qiang Kui Yang
Kou Qiang Kui Yang Jiu Bu Shou Lian
Kou Qiang Yan Zheng
Kou She Sheng Chuang
Kou Sheng Chuang
Lao Sou
Li Ji
Pi Fu Zhong Tong
Re Lin Se Tong
Se Tong
She Sheng Chuang
She Chong Yao Shang
Shen Re
Shou Xin Re
Sou Tong

Wu Ming Zhong Tong
Xiao Bian Pin Shu Duan Se
Xiao Bian Se Tong
Xiao Bian Tong
Xiao Er Gan Mao
Xiao Er Gao Re
Xie Xie
Xie Xie Ji Po
Xie Li
Xie Zhong Teng Tong
Yan Bu Chong Xie
Yan Bu Hong
Yan Bu Hong Zhong
Yan Bu Zhong
Yan Bu Zhong Tong
Yan Hong Zhong Tong
Yan Hou Bu Li
Yan Hou Teng Tong
Yan Hou Zhong
Yan Hou Zhong Tong
Yan Hou Zhong Yong
Yan Yong
Yao Di Suan Tong
Yong Zhong
Yong Zhong Chuang Yang
Zhong Tong
Zhong Ying Zuo Tong
Re Lin
